# Environmental Justice and Systems Analysis for Air Quality Planning in the Port of Oakland in California

**DOI:** 10.1021/acs.est.3c07728

**Published:** 2024-05-02

**Authors:** Fiona Greer, Ahmad Bin Thaneya, Arpad Horvath

**Affiliations:** Department of Civil and Environmental Engineering, University of California, Berkeley, Berkeley, California 94720, United States

**Keywords:** equity, supply chains, human health, exposure, fine particulate matter, freight, intake, inhalation intake, embodied impacts, shipping, transportation

## Abstract

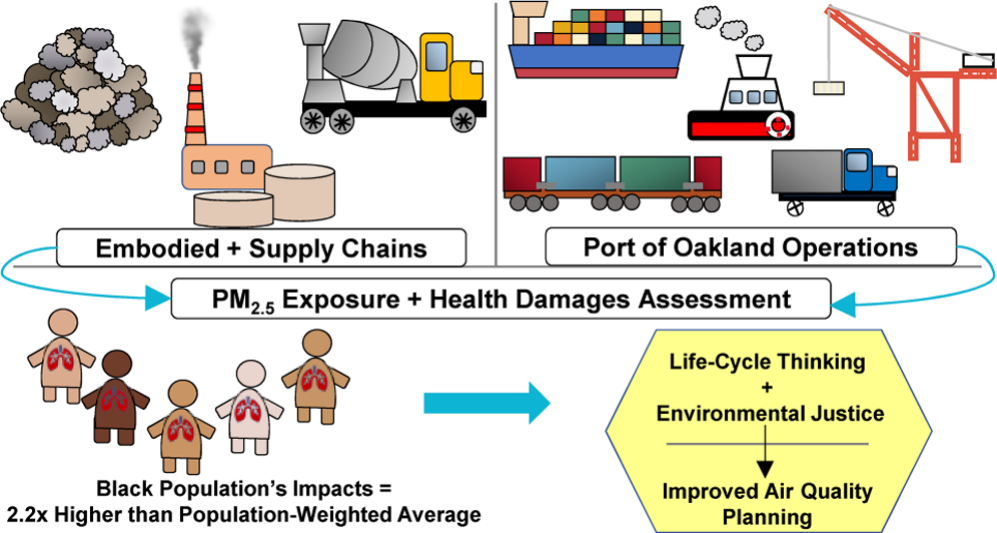

Many frontline communities experience adverse health impacts from living in proximity to high-polluting industrial sources. Securing environmental justice requires, in part, a comprehensive set of quantitative indicators. We incorporate environmental justice and life-cycle thinking into air quality planning to assess fine particulate matter (PM_2.5_) exposure and monetized damages from operating and maintaining the Port of Oakland, a major multimodal marine port located in the historically marginalized West Oakland community in the San Francisco Bay Area. The exposure domain for the assessment is the entire San Francisco Bay Area, a home to more than 7.5 million people. Of the more than 14 sources included in the emissions inventory, emissions from large container ships, or ocean-going vessels (OGVs), dominate the PM_2.5_ intake, and supply chain sources (material production and delivery, fuel production) represent between 3.5% and 7.5% of annual intake. Exposure damages, which model the costs from excess mortalities resulting from exposure from the study’s emission sources, range from USD 100 to 270 million per annum. Variations in damages are due to the use of different concentration–response relationships, hazard ratios, and Port resurfacing area assumptions. Racial and income-based exposure disparities are stark. The Black population and people within the lowest income quintile are 2.2 and 1.9 times more disproportionately exposed, respectively, to the Port’s pollution sources relative to the general population. Mitigation efforts focused on electrifying in-port trucking operations yield modest reductions (3.5%) compared to strategies that prioritize emission reductions from OGVs and commercial harbor craft operations (8.7–55%). Our recommendations emphasize that a systems-based approach is critical for identifying all relevant emission sources and mitigation strategies for improving equity in civil infrastructure systems.

## Abstract 2

People of color and low-income groups bear a disproportionate exposure burden to fine particulate matter from civil infrastructure systems. This study presents an application of an exposure assessment and human health damages framework on a major marine port, illustrating how air quality planners can standardize environmental justice and life-cycle thinking to mitigate the impacts from civil infrastructure systems.

## 1 Introduction

There is increasing recognition of the undue burdens that communities of color, those experiencing economic disparity, and other marginalized groups face from the construction and operation of the civil infrastructure systems that support our society. Many of these frontline communities have adverse health impacts and other negative outcomes due to living in proximity to high-polluting industrial sources.^1^ The first and sometimes the strongest and only voices raising concerns about inequity in pollution exposure have been the communities themselves. Exposure refers to the amount of pollution a person or group of people experience in a specific geographic location often over a designated period of time; exposure is a function of duration and concentration.^2^ Affected communities have often self-organized in efforts to demand that regulatory bodies offer remediation from pollution.^3^ This effort has sparked a larger environmental justice movement where now local, state, and the federal governments are considering inequities in the context of new project developments, existing operation of polluting sources, and planning for major investments of climate change funding.^4,5^ It is important to ensure that environmental justice considerations, particularly if they are going to be standardized into a government action framework, be reflective of these community group efforts and include a comprehensive set of quantitative indicators to measure progress. It is useful to see how this approach might be enacted with a case study so we can identify suggestions for future implementation of air quality planning approaches. West Oakland, a neighborhood in Oakland, California, is a compelling and appropriate case study for examining the effectiveness of this approach.

West Oakland is a well-studied area of air pollution, largely due to efforts from community groups to draw attention to the pollution sources. Bounded by freeways, rail lines, and a major marine port, West Oakland is a community of historical, social, and political significance within the city of Oakland and the San Francisco Bay Area.^6^ Home to a primarily low-income Black and Latino neighborhood, West Oakland has a documented history of poor air quality, and its residents experience disproportionally worse health outcomes and lower life expectancies relative to residents of surrounding Alameda County.^7^ When compared to the rest of the county, residents of West Oakland experience 21%, 33%, and 62% higher incidents of premature mortality due to cancer, heart disease, and stroke, respectively.^7^ Emergency hospital visits and hospitalizations for asthma are 74% and 88% more frequent, respectively, for West Oakland residents than for the average Alameda County resident.^7^ West Oakland residents’ disproportionate adverse health outcomes are partially attributable to the proximity of high-polluting transportation and industrial sources, which have led to higher relative exposures to diesel particulate matter, fine particulate matter (PM_2.5_), other cancer-inducing toxic air contaminants, and noise.^7^

High pollution in West Oakland is in part attributable to historical redlining practices. Beginning in the 1930s, the Home Owners’ Loan Corporation, along with local real estate authorities, created a system of “redlining” property areas ostensibly to create a framework for categorizing areas of risk for mortgage loans.^8^ In reality, areas with higher populations of people of color, such as West Oakland, were deemed “Fourth Grade” or “Hazardous”, which depressed housing values and continued to make the land attractive for locating industrial facilities along with the facilities that existed prior to the practice.^8,9^ Historical redlining practices have been shown to be associated with present-day pollutant exposure disparities, where pollution levels are over 50% higher in redlined zones compared to higher preferentially graded neighborhoods.^10^ Current pollution sources in West Oakland include shipping and freight activity at the Port of Oakland, vehicle traffic from Port operations and adjacent Interstate highways 880, 980, 580, and 80, manufacturing plants, and freight rail activity. These sources are contributing to exacerbated environmental and health impacts in the community.^7^

The disproportionate exposure burden faced by the West Oakland community has led the state’s air pollution regulatory body, the California Air Resources Board (CARB), to designate the area as a candidate for the Community Air Protection Program.^11^ Participation in the program entails community-led development of an emission reduction program to mitigate exposure from freight, trucking, industrial manufacturing, and port sources. The community-led approach is a new effort by the state government to identify improved practices for air quality planning, as prior research and lived experience indicates that community members are often the best-suited for identifying effective mitigation solutions.^3^ The Community Air Protection Program limits its focus to sources within the boundaries of the community and does not account for relevant upstream sources that can be impactful on community members’ exposure burdens.

We apply an analytical framework to determine potential gaps in academic, government, and community-led efforts to identify the most important emission sources and mitigation strategies for West Oakland, focusing our efforts on the operation and maintenance activities associated with the Port of Oakland. We apply life-cycle thinking, exposure assessment, and health damages modeling to comprehensively quantify the exposure disparity caused by the Port of Oakland. Examining a full scope of emissions, sources, and attributable impacts are necessary for guiding future policy decisions aimed at making our civil infrastructure systems more equitable. It is vital that policy decisions rely on analysis centered on life-cycle, systems-level thinking as incorporating life-cycle phases (e.g., material manufacturing, supply chains) has been shown to significantly increase criteria air pollutant emissions estimates from goods movement in marine ports.^12^ Critical research questions that this work intends to answer include:

1) Using the 2020 (the latest) emissions inventory for the Port of Oakland, excluding emissions from cruise ships confined to the Port because of COVID-19 restrictions, what is the baseline PM_2.5_ exposure, in terms of intake, from a typical marine port’s operations and routine maintenance? How does exposure affect demographic groups by race/ethnicity and median income?
2) How do emissions and exposure from the Port’s operations compare to emissions and exposure from some of the fuel supply chains and materials used in maintaining the Port?
3) How effective are mitigation strategies in reducing exposure from specific sources? Which mitigation strategies are the most important to consider?
4) What are the PM_2.5_ exposure damages, based on value of a statistical life (VSL), for the Port of Oakland?

The overarching objective in this research is to apply existing air pollution assessment methods with life-cycle thinking that can then complement community-led efforts in quantifying the environmental justice implications from operating and maintaining civil infrastructure systems. Specific objectives in this research are to map the current PM_2.5_ exposure burden from the Port of Oakland’s annual operations and routine maintenance onto the system boundary of the San Francisco Bay Area, explore effective PM_2.5_ exposure mitigation strategies, and offer a reasonable estimate of the economic harm caused by the Port’s pollution.

The remainder of the article is organized into a literature review section (2. West Oakland and Its Air Quality Research Roots), a methods section (3. Methods), a results section (4. Port of Oakland: PM2.5 Exposure Impacts and Pathways for Mitigation), and a discussion and conclusion section (5. Discussion and Conclusions). In Section 2, we cover the history of relevant academic and community-led research efforts on quantifying West Oakland’s and the Port’s pollution sources and their impacts. Section 3 describes our exposure assessment and economic damages methodological framework. Section 4 details the baseline exposure results as well as the results of various targeted mitigation strategies. Section 5 discusses the implications of the study’s results in centering and quantifying environmental justice in the air quality planning of our civil infrastructure systems, and in expanding the system boundary of analysis to reflect life-cycle thinking by including relevant upstream sources.

## 2 West Oakland and Its Air Quality Research Roots

A key figure in West Oakland’s pollution story is the Port of Oakland which is the ninth busiest container port in the United States and one of the four busiest ports on the West Coast.^13^ The movement of freight is an essential cornerstone of the United States economy. Marine ports, which typically include intermodal freight facilities such as trucking and railyards, facilitate the movement of, on average, $2.7 trillion in imports and exports annually.^14^ The goods supply chain is sensitive to shipping container port operation; disruptions caused by the COVID-19 pandemic led to backlogs at multiple U.S. ports leading to increases in pollution in port-adjacent communities.^15,16^ A major driver behind the industrialization and high traffic in the West Oakland area is the Port of Oakland, which is a documented source of pollution within the San Francisco Bay Area and of special concern as a significant contributing source to pollution within the West Oakland community.^17^

The Port of Oakland has been an area of interest not only as a pollution source that the residents and community members are working to mitigate, but also as a research area for testing mitigation and pollution monitoring strategies. Most prior research on the Port, of which a comprehensive literature review is presented in the Supporting Information**(SI)** document, has focused on drayage trucks which are the heavy-duty diesel-powered trucks that carry shipping containers out of the Port to their next destination, such as a wholesale distribution center. The consensus from prior studies indicates that drayage truck emissions are a major cause of the health and environmental impacts seen in the West Oakland area, and that regulatory efforts toward their mitigation are required.^18−22^

Community-led research has acted as a major driver in reducing exposure impacts in West Oakland. Since the early 2000s, residents of West Oakland have organized to identify environmental issues in the community, measure indicators of these pollution impacts, and translate these findings into effective policy countermeasures to reduce pollution impacts in the community.^23^ One study analyzed the changes in exposure associated with the rerouting of the Cypress Freeway, which originally ran through West Oakland before community organizers and residents successfully advocated for a street-level boulevard built in its place after the 1989 Loma Prieta earthquake, and how these changes manifest themselves in benefits to different demographic groups in West Oakland.^24^ The effort of rerouting the Cypress Freeway reduced annual nitrous oxides (NO_*x*_) and black carbon (BC) concentrations by 38% and 25%, respectively, along the new parkway. However, the benefits of these reductions were not experienced by the original residents along this freeway, which saw a reduction in Black populations by 28%. The study’s author attributes the shift in demographics to an 184% increase in property value along the newly built street-level boulevard. This research shows that while community advocacy efforts can result in environmental benefits for minority and low-income groups, further policy efforts need to ensure that these communities are protected such that they are not displaced from these areas due to increases in property values that come from the implemented environmental changes.

There have been other recent efforts to analyze the efficacy of partnerships between community-led and community-based organizations and academic partners in identifying, assessing, and addressing environmental issues in West Oakland. A case study explored a community organization’s advocacy efforts in the reduction of disproportionately high diesel emission exposures through truck route ordinance in the West Oakland area.^25^ Despite their efforts, the community-led group could only manage weak enforcement of their truck-control policies; however, they did gain increased political visibility and higher participatory engagement in environmental decision-making at the local and regional levels.

Participatory-research in West Oakland led to the development of the West Oakland Community Action Plan, a collaboration between the Bay Area Air Quality Management District (BAAQMD) and the community-based West Oakland Environmental Indicators Project (WOEIP).^7^ The initiative seeks to improve air quality conditions for West Oakland residents through community-supported local mitigation plans. The ongoing effort tracks and reports the state of environmental health impacts, sets mitigation targets, and tracks implementation and progress toward mitigation goals.

Outside of the efforts organized by West Oakland community groups and through AB 617,^26^ there are fewer research efforts that investigate the cumulative effects of all emission sources from the Port of Oakland, or attempt to connect pollution from the Port to a measurable impact such as increased risk of mortality. A study investigating the effects of regulating the heavy fuel oil for port container ships in the Bay Area concluded that regulations to reduce the high sulfur content of the fuel led to reductions of ambient PM_2.5_ concentrations by 3.2%.^27^ One study used West Oakland neighborhoods near the Port to demonstrate that mortality from pollution-attributed risks can vary at fine spatial scales within an individual city.^28^ There is a significant gap in exploring how all sources within the system boundary of the Port impact the community at large and in specific racial and socioeconomic groups in particular. Additionally, there are no existing studies that examine the significance of exposure burdens from upstream sources related to Port maintenance and operation.

## 3 Methods

### 3.1 Methods Overview

**Figure 1:**
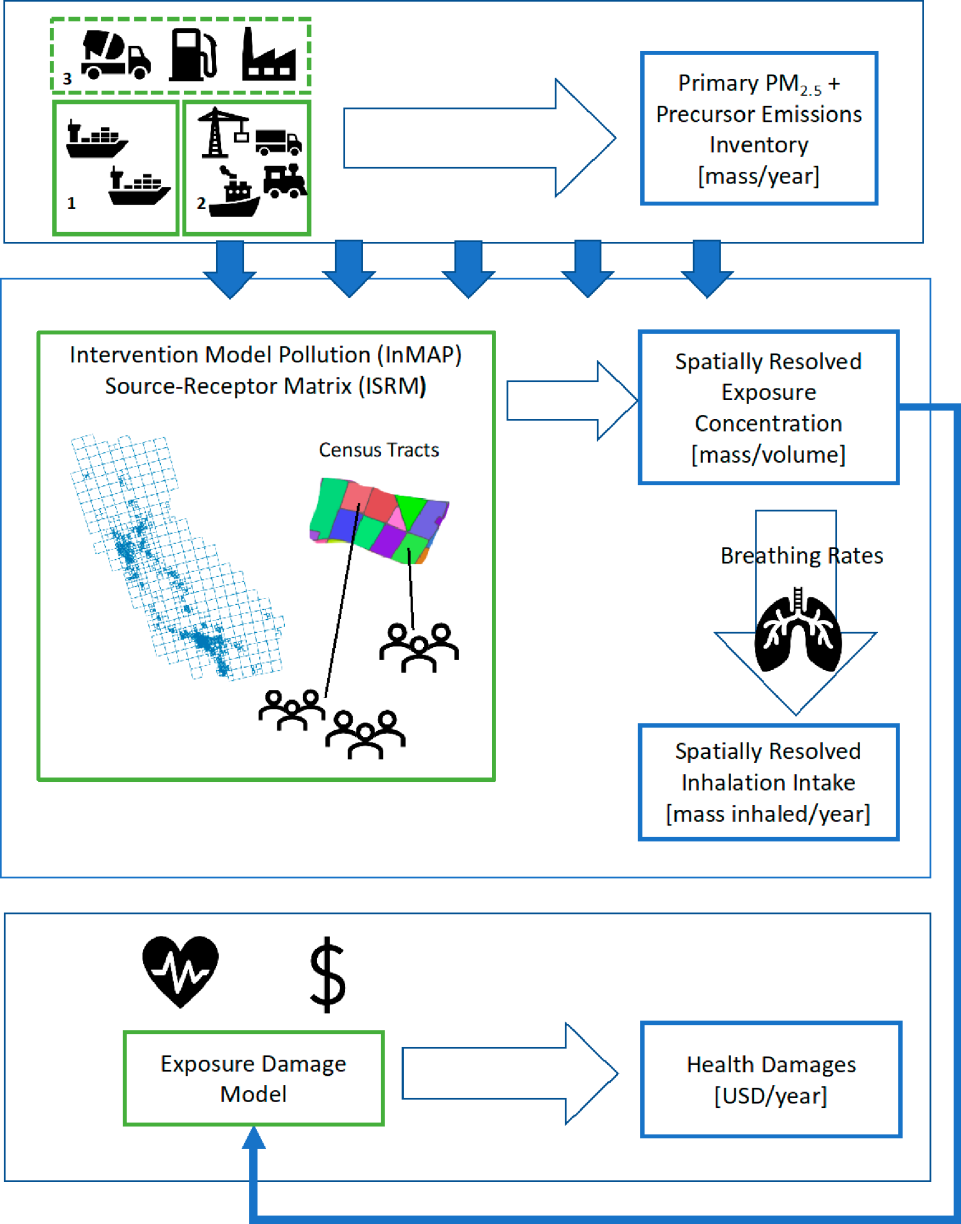
Summary of methodological steps. The primary PM_2.5_ and precursor emissions inventory consists of: (1) direct emissions from ocean-going vessels (OGVs); (2) direct emissions from in-harbor ships and intermodal port operations; (3) embodied emissions from port surface material production/delivery and drayage fuel. Census tracts within the exposure domain, along with the emission inventory, are fed into a reduced-complexity air quality model to produce spatially resolved ground-level average exposure concentrations. Average population breathing rates are used to quantify the amount that each person within the exposure domain inhales in a year. Health damages from excess mortalities are estimated using the spatially resolved average exposure concentration values.

We present an exposure assessment of and estimated health damages from primary PM_2.5_ and secondary precursor emissions associated with the operation and maintenance of the Port of Oakland. Figure 1 summarizes the methodological steps in conducting the exposure assessment and health damages estimation. The methodology for exposure modeling, which follows Bin Thaneya et al.,^29^ first involves developing a spatially resolved emissions inventory. Changes to ground-level PM_2.5_ concentrations due to port-related emissions are then estimated using a reduced-complexity air quality model.^30^ The emissions inventory, which comes from both a 2020 report commissioned by the Port of Oakland as well as a report from the West Oakland Environmental Indicators Project,^26,31^ is fed into the Intervention Model Pollution (InMAP) Source-Receptor Matrix (ISRM).^32,33^ The ISRM calculates marginal changes in ground-level PM_2.5_ concentrations which are then used to determine resulting exposure, or inhalation intake, from the spatially resolved emissions inventory. Census tract data for the exposure domain of the San Francisco Bay Area are applied to investigate PM_2.5_ exposure concentration and inhalation intake values by mitigation strategy and demographic group.^34^ We then take the average exposure concentrations and calculate human health damages using an exposure damages model. The model quantifies incidences of premature mortality due to increases in PM_2.5_ concentrations from Port emissions using established concentration–response functions,^35,36^ and the EPA recommended value for statistical life (VSL) metric^37^ is then used to convert the excess mortality figures into monetary damages. It should be noted that these methods are applicable for locations outside of California and the United States as long as one has a spatially resolved emissions inventory, population data, and a reduced-complexity air quality model.

### 3.2 Emissions Inventory

**Figure 2:**
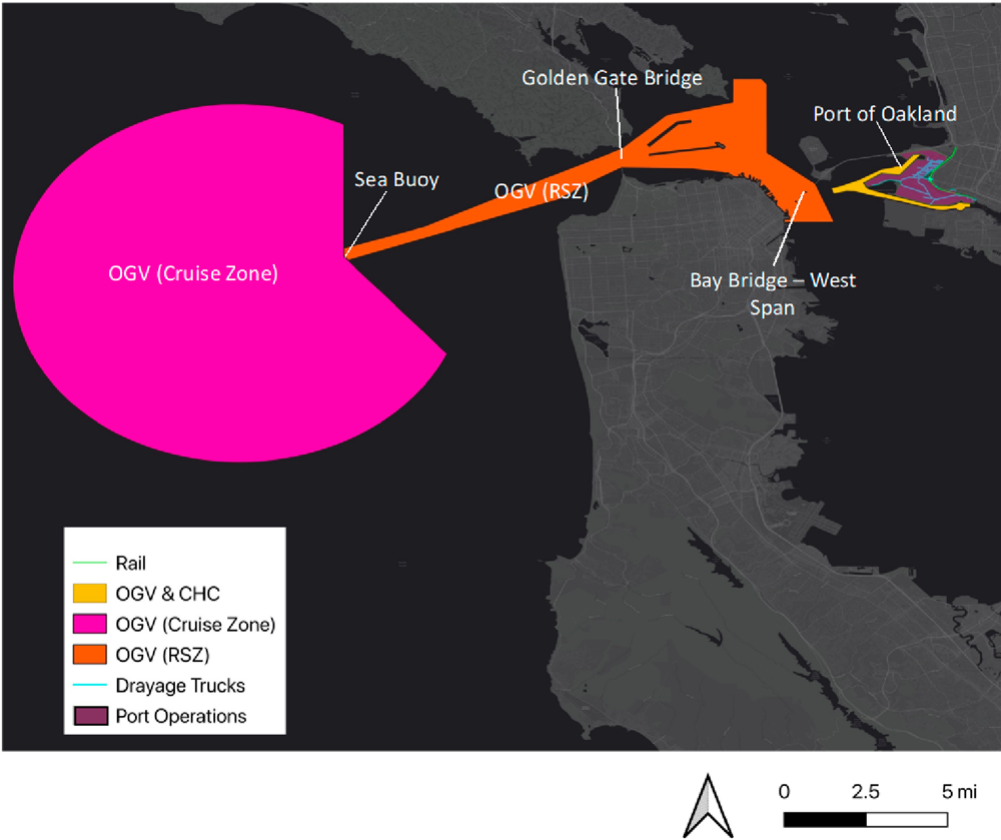
Scope of operational sources from Port of Oakland accounted for in study.

**Figure 3:**
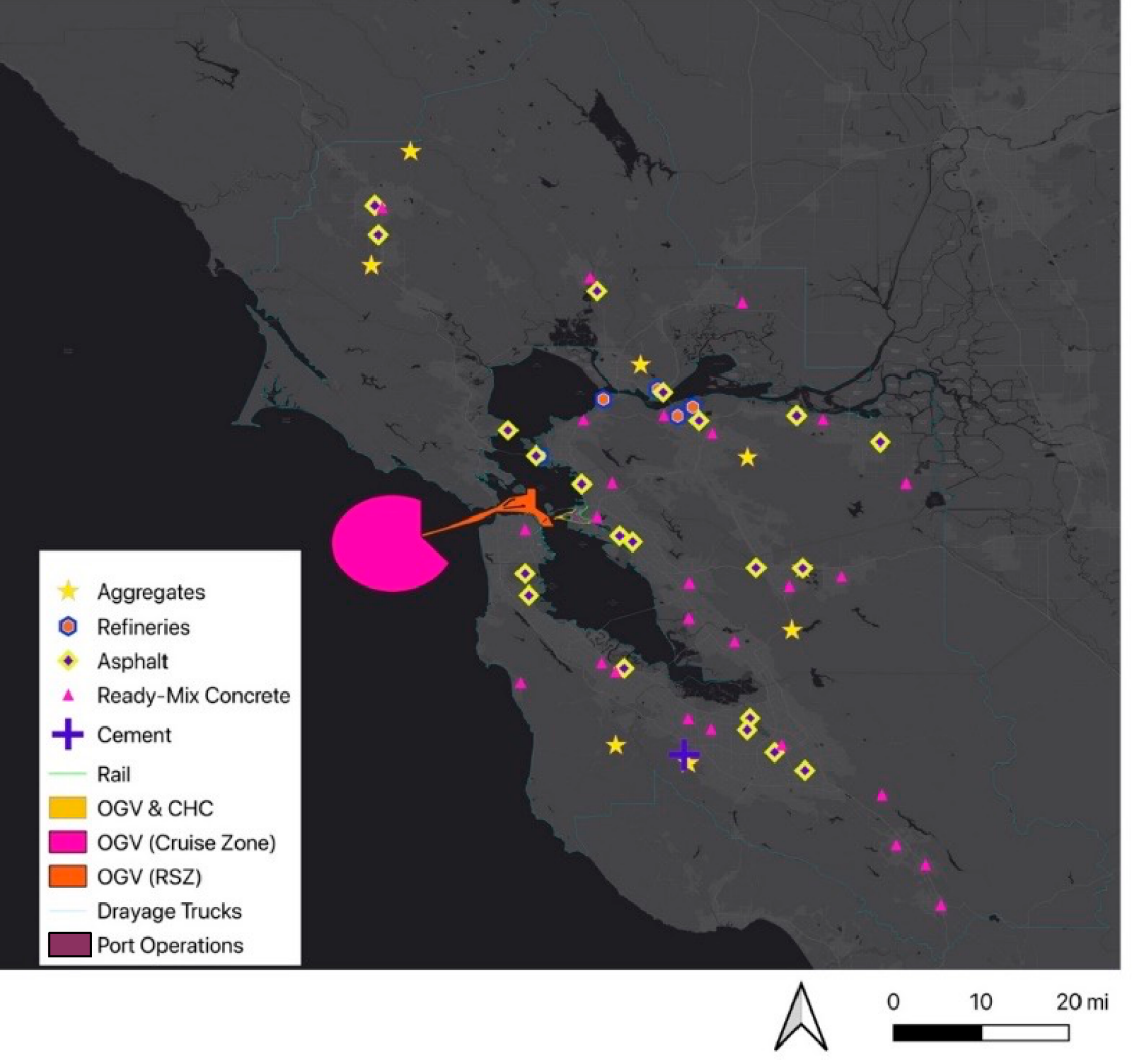
Location of material facilities within the Bay Area relative to the Port of Oakland. The closest ready-mix concrete production facility is used as the representative supplier of concrete used in the annual maintenance of the Port.

The emissions inventory, depicted in Figures 2 and 3, consists of both direct emissions from (1) ocean-going vessels (OGVs), or large container ships, entering the Bay Area and anchoring at the Port of Oakland and (2) both smaller ships that assist OGVs within the Port’s harbor and from intermodal operations at the Port itself (e.g., cargo handling equipment, rail), as well as (3) embodied emissions from the production of materials used in maintaining the structural integrity of the Port’s surface area, fuel needed to deliver the materials to the Port, and fuel used by the drayage trucks operating within the Port boundaries (i.e., idling within the Port terminal and driving from the Port terminal to three freeway entrances). **Table S1** in the SI document lists and describes in detail the more than 14 distinct emissions sources included in the emissions inventory.

In developing the embodied emissions inventory, we assess how much materials would be needed each year to maintain the structural integrity of the Port’s surface. The area of the Port is just over 2 square miles (5.3 km^2^).^38^ Information about the design of the Port’s surface is limited to a report on the construction of two of the Port’s berths from the early 2000s.^39^ To account for maintenance emissions and induced exposures, we assume that the berth design is an approximate representation of the entire surface area for the Port of Oakland. The design encompasses an approximately 4-in. (10 cm) surface layer of concrete (assumed to be normal strength) on top of a 1-in. (2.5 cm) layer of aggregate, a 3-in. (7.5 cm) layer of asphalt, and an almost 19-in. (47 cm) layer of compacted aggregate base. It is assumed that for maintenance purposes, around 2 to 5% of the total Port area would be reconstructed each year, but that new material would not be brought in for the compacted aggregate base layer. The assumption that new materials would not be brought in for the compacted aggregate base layer follows procedures typical of maintenance and resurfacing of highways,^40^ which are closely analogous in design to the Port’s pavement structure. While the assumption on the range of how much of the Port’s pavement area is maintained each year is intended to mitigate uncertainty, limitations with this assumption are discussed in the Discussion and Conclusions section. PM_2.5_ emission factors are based upon emission rates from material plants within the Bay Area that have CARB annual emissions data. It is assumed that all concrete comes from the closest available ready-mixed concrete plant. All other material needs are sourced from respective plants within the exposure domain.

The inclusion criteria for emission sources within our system boundary vary for several reasons. The first reason is due to the availability of quality emissions inventory data. The direct emissions inventory data developed by the Port and WOEIP is the most detailed of its kind, including a breakdown of emissions by individual operating sources within the Port’s boundaries. There are a lack of data regarding the routes and final destinations of goods movement sources (e.g., rail cars, drayage trucks) once they leave the Port and so emissions from goods movement directly outside of the Port’s physical boundaries (which in the case of the drayage trucks are anywhere past the freeway entrances) are excluded. A fundamental goal of this research is to expand the scope of emissions that Port stakeholders consider and decide to control. Therefore, we account for embodied emissions associated with the Port’s operation and maintenance. Embodied emissions are associated with the raw material extraction and production, transportation, manufacturing/construction, maintenance, and end-of-life processes for a product, project, or system. With the rise in the decarbonization of energy sources that lead to operational emissions, the embodied emissions of the built environment (e.g., from steel and cement use) are expected to become even more significant contributing sources of emissions in the future,^41−43^ signaling the need for their inclusion in environmental health assessments. We focus our embodied emissions on the likely sources that would be located and efficiently modeled within the exposure domain including local material production facilities (e.g., the rapid setting of concrete necessitates the proximity of batch plants with projects) and fuel, produced at refineries within the exposure domain, used to deliver resurfacing materials to the Port and to power on-site drayage truck operations. Embodied emissions from the goods that pass through the Port are thus excluded because it is likely that they are not manufactured within our system boundary. We also exclude direct emissions from construction activities to perform the Port surface maintenance and embodied emissions from OGV and off-site drayage truck fuel consumption primarily due to a lack of reliable data to accurately model these sources. Future research aimed at tracking and modeling these excluded sources would help to assess the entire scope of the Port’s emissions more holistically. We consider the implications of these system boundary decisions on the overall exposure results within the Discussion section.

### 3.3 Exposure Assessment Model

Exposure assessment is a framework for determining the impact air pollution has on a population. We employ two impact indicators in this study: (1) ground-level average exposure concentrations (units of micrograms of PM_2.5_ per cubic meter of air) and (2) annual inhalation intake (units of grams of PM_2.5_ inhaled by the exposed population over a year). The two indicators offer a more comprehensive picture of the impacts from the sources of Port pollution. As discussed in more detail in subsequent sections, average exposure concentrations support monetary health damage and equity analyses while inhalation intake presents the annual, cumulative impact that pollution poses. Both indicators are estimated for an exposure domain, or population of interest. In this study, the exposure domain is the nine-county San Francisco Bay Area region which is home to more than 7.5 million people.^34^ Inhalation intake is quantified by multiplying average exposure concentration by an average breathing rate of 15 m^3^ per person per day.^44^ Note that the inhalation intake for the entire exposure domain is found by multiplying the per-person intake in each census tract by the total number of people within that tract and summing those values for the entire exposure domain.

The spatially resolved inventory of primary PM_2.5_ and secondary formation of PM_2.5_ from NO_*x*_, volatile organic compounds (VOCs), sulfur dioxide (SO_2_), and ammonia (NH_3_) precursors that are emitted in the year 2020 is input into the ISRM, a linearized version of InMAP. ISRM models marginal changes in ground-level concentrations using a source-receptor matrix with grid cells that vary in size depending upon the area’s population density. Grid sizes are as small as 1km × 1km in and as large as 48km × 48km in high and low population density areas, respectively.^30^ InMAP’s use of variable grid sizes reduces the runtime of multiscenario model running by allowing for finer concentration gradient calculations only in areas with high population numbers. In our study, the source locations of pollution, which are geospatially joined to GIS shapefiles, are the physical boundaries of Port of Oakland and its shipping operations (Figure 2) and the sites of material production facilities/refineries and delivery routes (Figure 3). The receptors of interest are zones where people live. Population demographic and location information come from census tract data from U.S. Census Bureau statistics.^34^ Census tracts vary in population count, but the 25th and 75th percentile population counts in all tracts only range between 3,500 and 6,000 people. Overall, there are 1576 census tracts included in the exposure domain. We elected to use InMAP/the ISRM, which models the physical transport and chemical formation of pollutants using 2005 WRF-Chem based meteorological data, as our mechanistic air quality model because of its efficiency^32,45^ and applications to exposure domains of similar scale.^29,46−48^ InMAP has also been used outside of the United States,^49−51^ making the methodological steps presented in this research adaptable for other locations. Despite some limitations in the InMAP/ISRM (e.g., based on 2005 meteorological data, use of annually averaged input data, parametrization of some chemical relationships, underestimation of particulate sulfate and overestimation of particulate ammonium formation), it has been shown to have similar performance to other reduced-complexity air quality models and is within published air quality model performance criteria used to quantify monetized health damages.^33,52−54^

### 3.4 Equity Analysis

Using demographic data within each census tract, we determine exposure impacts for each population group. The equity analysis examines how air pollution from the sources studied within our exposure domain impacts different race/ethnicity groups and different socioeconomic groups. The race/ethnicity categories (Black or African American, White, American Indian/Native American or Alaska Native, Asian, Hispanic or Latino, Native Hawaiian or Other Pacific Islander) are designated by the U.S. Census Bureau. Socioeconomic differences are investigated by comparing impacts among different mean income quintiles (Q1 = < 20% lowest mean income, Q2 = 20–40%, Q3 = 40–60%, Q4 = 60–80%, Q5 = > 80% highest income) which are also established from U.S. Census Bureau data.

The equity impacts from the air pollution are quantified by estimating the exposure disparity, an emerging environmental justice indicator.^10,36,55−57^ Exposure disparity refers to the difference in exposure that one group experiences relative to the average exposure that all groups experience. The absolute exposure that each group experiences is calculated as the population-weighted average concentration exposure for that specific group (e.g., the average of all exposure concentrations for all people designated as Hispanic in the census tracts within the exposure domain). Population-weighted average concentrations for demographic or income group ***d*** are calculated using **(**1**)**.
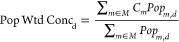
1where:

*C*_*m*_: PM_2.5_ concentration in census tract ***m***

*Pop*_*m,d*_: population count of demographic or income group ***d*** in census tract ***m***

We define the relative exposure disparity of group ***d*** as the difference between the absolute population-weighted average exposure concentrations for group ***d*** (Pop Wtd Conc_d_) and the population-weighted average exposure concentration of the entire exposure domain (Pop Wtd Conc_t_), all divided by the population-weighted average exposure concentration (which represents the control group being compared against) as shown in **(**2**)**.
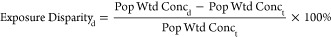
2

### 3.5 Mitigation Scenarios

A variety of realistic and future mitigation options to reduce the emissions footprint from Port of Oakland operation and maintenance are explored. Mitigation strategies are listed in Table 1.

**Table 1:** Port of Oakland Mitigation Strategies^a^

**Strategy Number**	**Description**
1	Truck 2045 Scenario
2	Truck Electrification
3	Rail Reduction (20%)
4	Trucking Reduction (20%)
5	OGV Cruise Reduction (20%)
6	OGV In-Harbor Reduction (20%)
7	CHC Reduction (20%)
8	OGV RSZ Reduction (20%)
9	OGV + CHC All Reduction (20%)
10	Port CHE Reduction (20%)
11	Port Other Reduction (20%)
12	Port Rail Reduction (20%)
13	Port + CHC All Reduction (20%)
14	Cement (20%)
15	RMC (20%)
16	Asphalt Reduction (20%)
17	Aggregate Reduction (20%)
18	Refineries Reduction (20%)
19	All Facility Reduction (20%)
20	OGV Harbor + CHC Emission Elimination
21	Combine All

a “OGV In-Harbor Reduction” refers to the following OGV operations: Shifts, Berths, Anchorage, Maneuvers.

The strategies are organized into three general categories: (1) future electrification rates of drayage truck operations and their fuel supplies; (2) reductions and total elimination, by unspecified means, of all other port operation emissions; (3) reductions, by unspecified means, of emissions from material production facilities. We also investigate a hypothetical scenario where all mitigation strategies are combined. We use emission factors from EMFAC for the Port of Oakland Class 8 Drayage (i.e., trucks that transport goods from marine port to their point of destination) vehicle type for an interim scenario that reflects levels of truck electrification in the year 2045^58^ and a future scenario where all drayage trucks are 100% electrified. Fuel supply chains for future diesel and electric-operated drayage trucks are calculated using the latest version of CA-GREET,^59^ which forecasts emission factors for diesel in the year 2045. Again, we assume that the future fuel and electricity will be sourced from providers within the exposure domain (i.e., fuel will be produced in the Bay Area refineries depicted in Figure 3 and electricity will be supplied from the region’s primary utility, Pacific Gas and Electric). It is assumed that electricity used in the 100% electrification scenario for drayage trucks is sourced entirely by nonemitting sources such as solar and wind.

It should be emphasized that the ISRM is well suited to assessing the efficacy of mitigation strategies because it inherently calculates marginal changes in exposure concentrations relative to a baseline emissions inventory. In this study, all marginal changes in exposure and inhalation intake from intervention strategies are calculated relative to the baseline, typical operations and maintenance for the Port of Oakland as defined in Section 3.2.

## 4 Port of Oakland: PM_2.5_ Exposure Impacts and Pathways for Mitigation

### 4.1 PM_2.5_ Exposure Results

**Figure 4:**
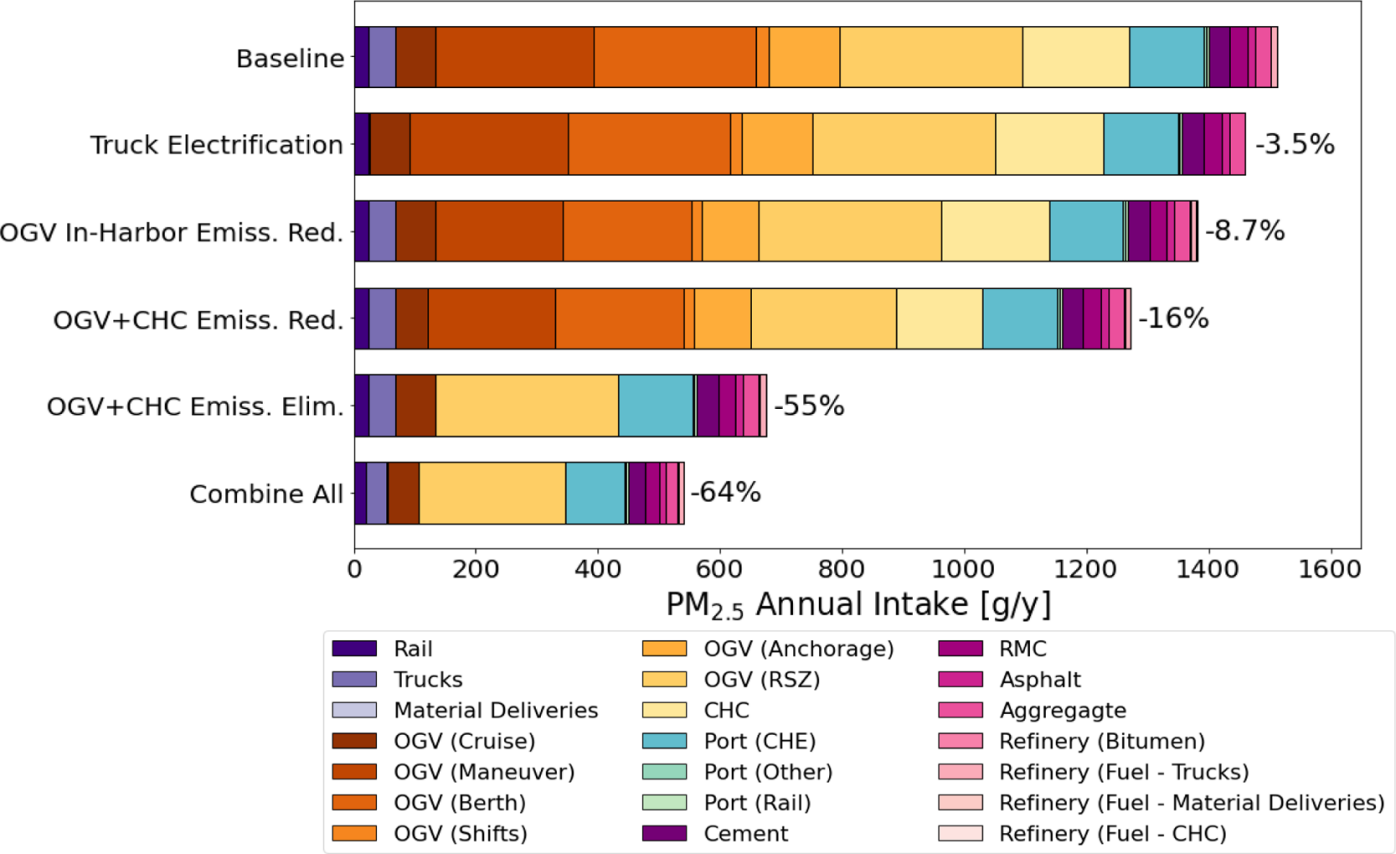
Annual PM_2.5_ intake values for the Port of Oakland for baseline and top-five mitigation strategies for the 5% resurfacing scenario. (OGV: Ocean-Going Vessels; RSZ: Reduced Speed Zone; CHC: Commercial Harbor Craft; CHE: Cargo Handling Equipment.

The average exposure concentrations under the 2% and 5% Port resurfacing scenarios are 0.035 μg m^–3^ and 0.037 μg m^–3^, respectively. Figure 4 depicts the annual baseline (no applied intervention) PM_2.5_ inhalation intake for the sources in our Port of Oakland emissions inventory in the 5% resurfacing scenario, in addition to the five most effective mitigation scenarios. The remaining scenario results are shown in the SI**(Figure S1)**. The annual inhalation intake from the Port of Oakland sources is 1510 g (1450 g) of PM_2.5_ per year in 5% (2%) resurfacing scenario. In the baseline condition when no mitigation strategy is applied, OGV sources dominate PM_2.5_ intake for the sources included within the study’s system boundary. Assuming 5% (2%) of Port surface volume gets maintained each year, all OGV sources account for 67 (71%) of annual inhalation intake. Within-harbor OGV operations (i.e., maneuvering, berthing, shifts, and anchorage) account for over 44% (46%) of annual PM_2.5_ intake in the 5% (2%) scenario. In-harbor OGV operations along with CHC operations (tugging) represents almost 55% (58%) of annual inhalation intake. In-Port trucking’s contribution is relatively small (3% of annual inhalation intake); however, the trucking result only accounts for drayage truck operation within the Port’s boundary and does not account for all trucking activity beyond the three freeway entrances adjacent to the Port in West Oakland. Inhalation intake from the embodied sources (material production facilities, material delivery, fuel production) represents 7.5 (3.5%) of annual inhalation intake.

Mitigation strategies aimed at reducing OGV and in-harbor sources yields larger reductions in annual inhalation intake values than any other mitigation strategy directed at mitigating individual sources. When all OGV harbor and commercial harbor craft (CHC) emissions are eliminated, annual inhalation intake is reduced by 55% to 58% to about 610 to 670 g of PM_2.5_ intake annually. Most other non-OGV and non-CHC related mitigation strategies yield modest reductions. The only strategy that results in moderate reductions apart from OGV-based reductions is a scenario where all in-port trucking operations are completely electrified (3.5% reduction). The largest reduction in annual inhalation intake occurs if all mitigation strategies are combined. This reduces annual inhalation intake by 64% to 68%. Depending upon how much port area is resurfaced each year as well as concentration–response functions and mortality hazard ratios, baseline exposure damages range from 100 to 270 million USD (in 2020 USD). Exposure damages for baseline and mitigation strategies are listed in **Table S4** in the SI.

**Figure 5:**
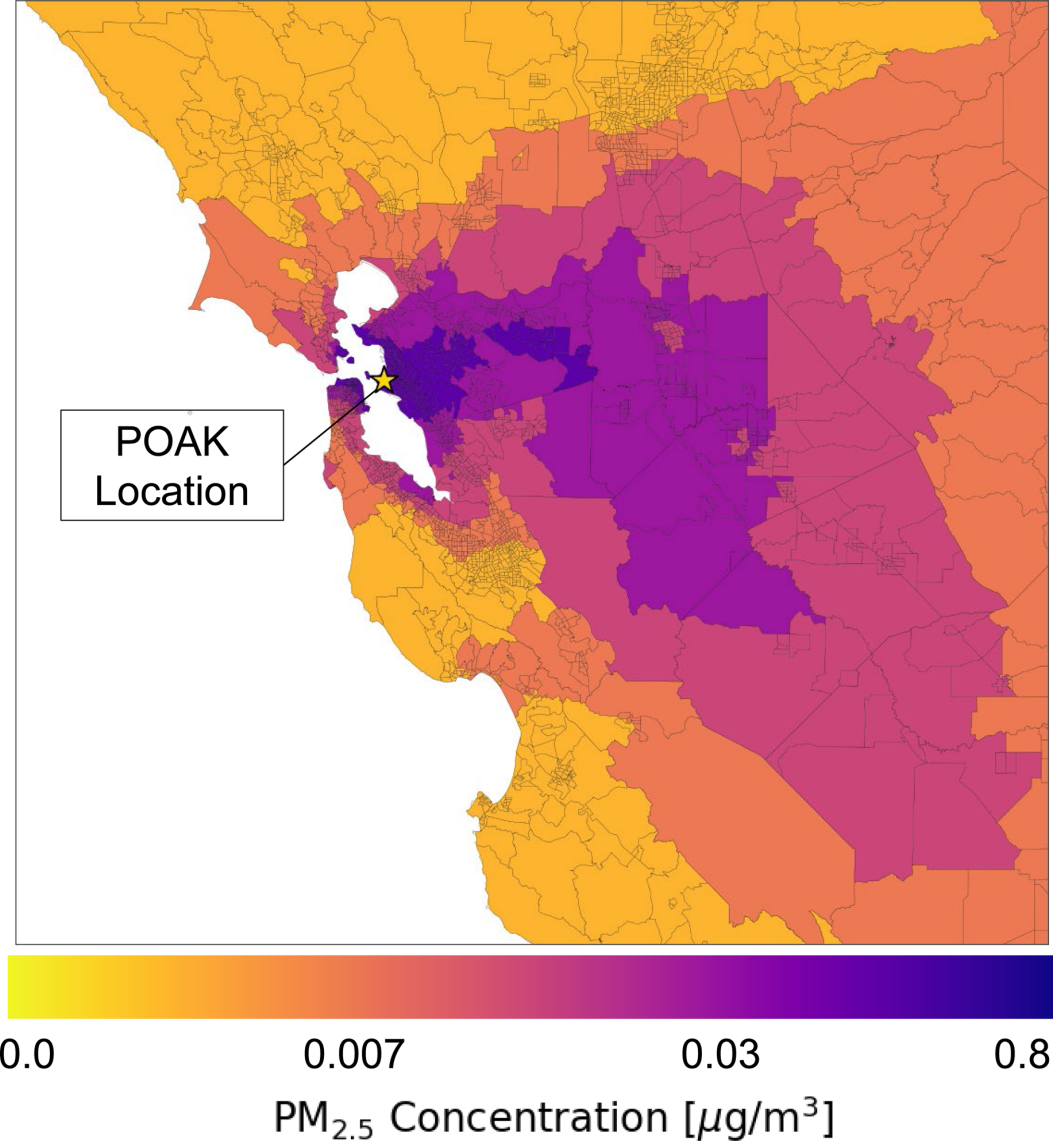
Spatial distribution of PM_2.5_ concentrations in the San Francisco Bay Area due to Port of Oakland related emissions.

Figure 5 depicts PM_2.5_ concentrations from the study’s emission sources as a function of distance from the Port of Oakland. Concentration gradients (**Figure S2** in the SI) are highest at distances <20km to the Port and concentrations quickly level-off beyond 20 km. We compare our InMAP generated PM_2.5_ concentrations to local PM_2.5_ concentration measurements from EPA AirData Air Quality Monitors^60^ to validate InMAP’s performance in the Oakland area. Given that our study only accounts for port-related emissions and concentrations measured at the monitoring sites are due to emissions from other sources as well, we obtain source apportionment data from several studies that quantify the contributions of port and marine shipping emissions to neighboring PM_2.5_ concentrations. The studies show that port and marine shipping emission contributions to local PM_2.5_ concentrations range between 1% – 17.^61−65^ 2019-based annually averaged PM_2.5_ concentrations from two Oakland monitoring stations were sourced (Oakland West and Laney College). Applying the port and marine shipping emissions proportion values yield measured cocentration ranging between 0.0310–1.32 μg m^–3^, with a mean concentration of 0.54 μg m^–3^ from the Oakland West monitoring station. The InMAP modeled PM_2.5_ concentration at the Oakland West location is around 0.56 μg m^–3^, showing generally reasonable agreement with the corrected measured data. Similarly, the corrected Laney College PM_2.5_ concentrations yield a range of 0.0296–1.26 μg m^–3^, with a mean concentration of 0.51 μg m^–3^. The InMAP-modeled PM_2.5_ concentration at the Oakland West location is around 0.52 μg m^–3^, again showing reasonable agreement between InMAP generated concentrations and measured concentrations in West Oakland. However, it should be noted that the level of agreement highly depends on the proportion of the concentrations that is attributed to port and marine shipping emissions.

### 4.2 Exposure Equity Considerations

**Figure 6:**
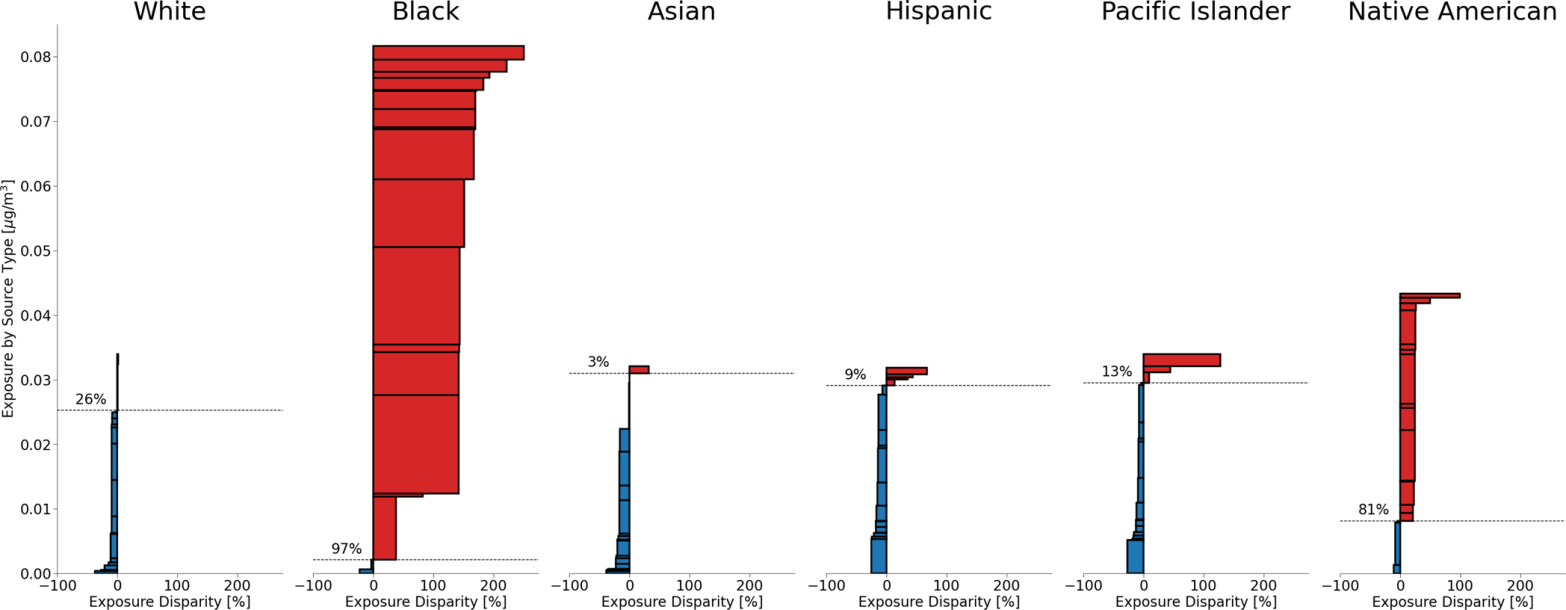
Absolute and relative PM_2.5_ exposure from Port of Oakland sources by racial demographic. The dashed horizontal line indicates the percentage of emission sources causing higher-than-average exposure for each group. Assumes 5% of the Port’s surface is reconstructed annually. The average relative exposure disparity is the weighted-average of all red and blue bar *x*-axis values (not shown in the figure). Average relative exposure disparities by demographic: White = −8%; Black = +120%; Asian = −13%; Hispanic/Latino = −15%; Native Hawaiian or Pacific Islander = −10%; Native American or American Indian = +17%.

**Figure 7:**
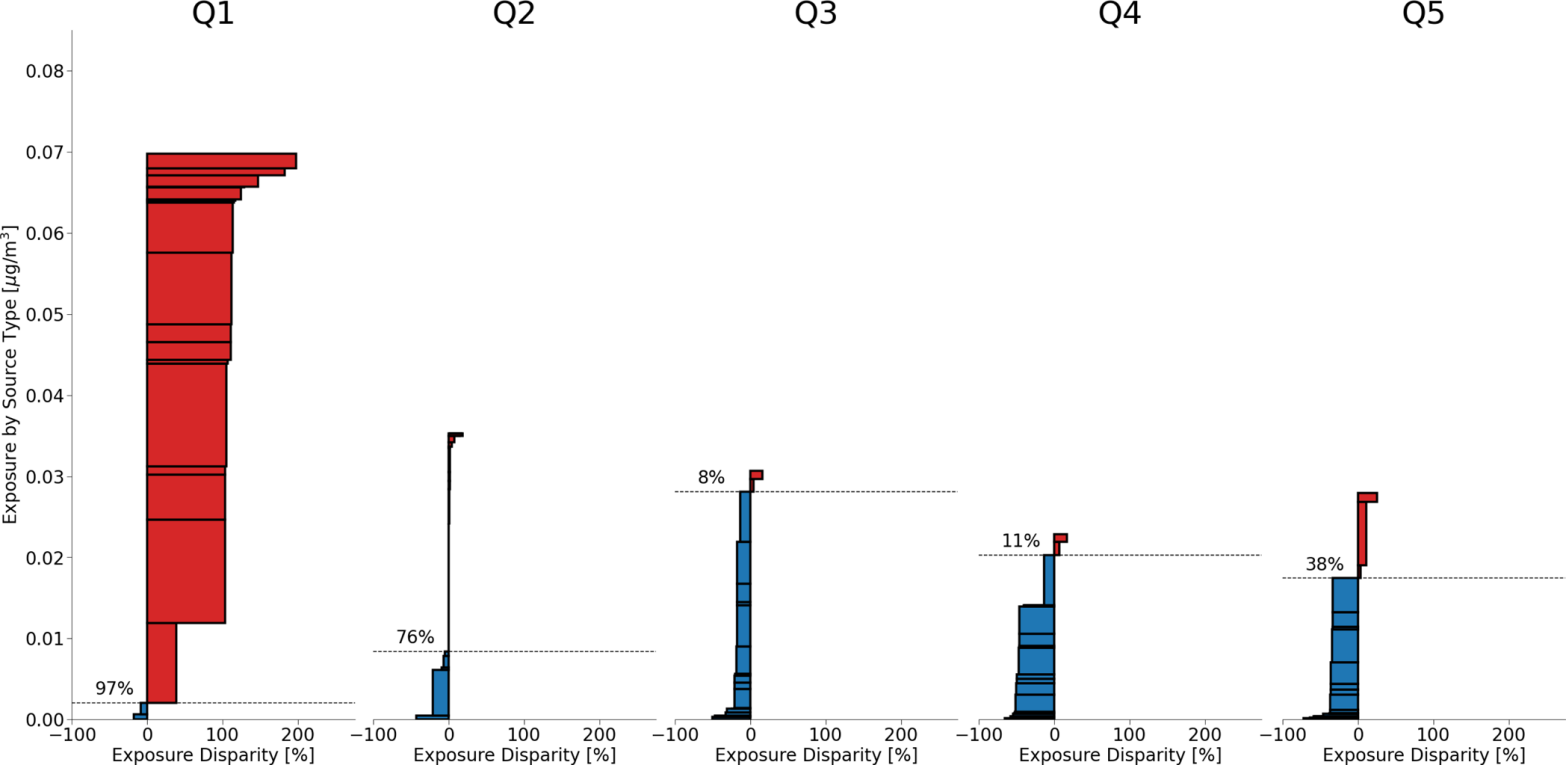
Absolute and relative PM_2.5_ exposure from Port of Oakland sources by income quintile. The dashed horizontal line indicates the percentage of emission sources causing higher-than-average exposure for each group. Assumes 5% of the Port’s surface is reconstructed annually. The average relative exposure disparity is the weighted average of all red and blue bar *x*-axis values (not shown in the figure). Average relative exposure disparities by income quintile: Q1 = +89%; Q2 = −4.4%; Q3 = −17%; Q4 = −38%; Q5 = −24%.

While the inhalation intake results in Figure 4 are the cumulative impacts the entire population within the exposure domain experiences in a year, exposure equity implications are best explored by examining averaged impacts. Figures 6 and 7 depict several important exposure equity impacts for each demographic and income group. The *y*-axis shows both the total average exposure experienced by each group (height of all horizontal bars) as well as the contribution of each emission source to the total average exposure (height of each individual bar). Each source bar is ranked according to its relative exposure disparity (*x*-axis), from the source type that the group experiences the most relative exposure disparity from to the least. The red-colored bars mean that a specific group experiences a greater-than-average relative exposure disparity for a specific emission source compared to the average exposure of that emission source for the entire population. The blue-colored bars reveal the opposite trend. The dashed horizontal line signifies the percentage of emission sources for which a group experiences a positive (i.e., greater than the entire population’s average exposure for a source) relative exposure disparity. For example, the Native American population experiences positive relative exposure disparities from 81% of all sources within the exposure domain. Finally, the weighted average of all red and blue bar *x*-axis values, results of which are not shown in the figures but described in the following text, tells us the average relative exposure disparity from all emission sources in the exposure domain for a group.

The exposure impacts disaggregated by demographic groups and income quintiles are starkly different. The Black population experiences an overwhelmingly greater-than-average PM_2.5_ exposure burden from the Port of Oakland sources, as shown in Figure 6. Under both resurfacing scenarios, the Black population's average relative exposure disparity is around 120%, meaning that a Black person within the exposure domain is 2.2 times more disproportionately exposed to the Port’s pollution sources than the general population. The Native American population also experiences a greater-than-average exposure disparity of 17%. The White, Asian, Hispanic/Latino, and Native Hawaiian or other Pacific Islander groups all experience lower-than-average exposure disparities from the Port of Oakland at −8%, −13%, −15%, and −10%, respectively. In terms of income (Figure 7), the only income quintile with greater-than-average relative exposure disparity is Q1 with 89% higher exposure than the population-weighted average.

The ranking of the source types in Figures 6 and 7, from the source that causes the greatest average exposure burden to the least average exposure burden, are located in **Tables S2** and **S3** in the SI. People of color within the Bay Area experience higher exposure disparities from the materials used in annual Port maintenance than the White population does. For example, the Black population’s highest exposure disparity comes from aggregate production (+250%). The White population experiences a lower-than-average exposure disparity of −37% from aggregate production (likely because much of the White population does not live near the material production facilities as the other populations do). Similar trends are observed for the income quintiles, with the lowest income quintile, Q1, being most disproportionately exposed to material production facilities. The only emission sources that the White population is disproportionately exposed to are emissions from OGVs in the cruise zone (+1.5%) and the reduced speed zone (+0.54%). Aside from OGV cruising, which occurs outside of the Golden Gate Bridge, all OGV operations disparately impact the Black population the most.

## 5 Discussion and Conclusions

### 5.1 OGV Operations

To achieve effective exposure reductions, policy measures need to target sources with the highest exposure damages. Based upon the emissions inventory studied in the specific exposure domain, OGVs appear to be the most significant source to control beyond trucking activities outside the Port of Oakland. OGV operations within the vicinity of the Port (i.e., the area between the San Francisco-Oakland Bay Bridge to the Port harbor) cause the highest impacts. The mitigation scenarios explored in this paper show that reducing or even eliminating in-harbor OGV activities by having them replaced with other less-polluting sources can yield significant exposure reductions. In terms of current regulatory efforts, CARB is in ongoing negotiations with Port authorities to address exposure impacts by implementing regulations to reduce the Port of Oakland’s diesel PM and NO_*x*_ emissions.^66^ CARB’s efforts began in 2007 with regulations for OGV for ports within California, including the Port of Oakland, with the goal of reducing PM and NO_*x*_ from OGV at berth operations by 80% by 2020. CARB is currently exploring how to expand the OGV regulations to include other vessel types (e.g., commercial harbor craft). CARB’s Comprehensive Truck Management Program^67^ also seeks to limit and phase out the model years for drayage truck engines which exceed emission thresholds.

### 5.2 Environmental Justice Implications

**Figure 8:**
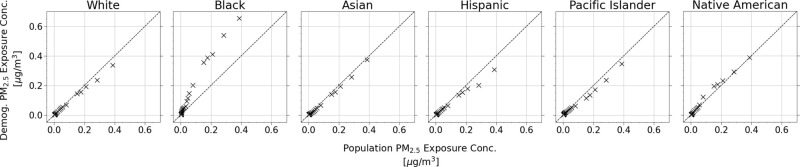
Q-Q plot of race/ethnicity demographic PM_2.5_ exposure concentrations relative to the population PM_2.5_ exposure concentration.

**Figure 9:**
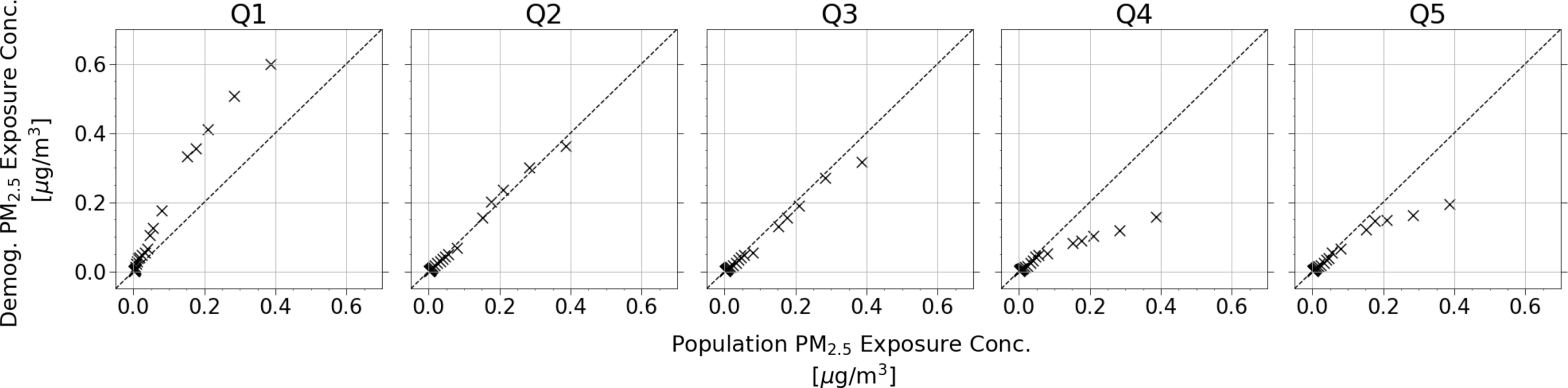
Q-Q plot of income quintile PM_2.5_ exposure concentrations relative to the population PM_2.5_ exposure concentration.

The extreme exposure disparities faced by the Black population and low-income groups due to the emission sources from the Port of Oakland highlight the importance of developing air quality planning and mitigation efforts for specific communities. To reduce the uncertainty in our conclusions about the air pollution disparities faced by specific demographics, we compared the distribution of exposure results per subgroup to the distribution of the population’s average exposure results in quantile-quantile (Q-Q) plots (Figures 8 and 9). Quantile data points are shown for percentiles in intervals of 10 as well as the top and bottom 5 percentiles. Data points lying above the 1:1 line signify that the demographic/income group is experiencing higher concentrations than the rest of the population at that percentile. Data points on the 1:1 line means they are equal, and data points below mean that they are experiencing lower average concentrations than the rest of the population. For the race/ethnicity demographics, the Black population has higher concentrations than the rest of the population, with the departure being more significant at higher percentiles. The disparity is more obvious by income, where Q1 sees much higher exposures while Q4 and Q5 are much lower.

The participation of community-based organizations in tracking pollution, setting reduction targets, and participating in policymaking, as seen in West Oakland, can be a catalyst for effective air quality and health benefits. This is especially true for communities that carry a large burden of the exposure disparities while being short-changed with respect to the economic benefits of that pollution source, which is an ongoing systematic issue in the United States.^36^ Despite the progress in West Oakland’s air quality, PM_2.5_ concentrations in certain census tracts can be as high as 0.8 μg m^–3^ due to the Port of Oakland sources alone, which is 20% of the San Francisco Bay Area’s annual average PM_2.5_ exposure concentration.^68^ The exposure concentrations along with total annual exposure damages range between 100 and 270 million USD (in 2020 USD), which indicates that more effort for mitigation remains essential. The novelty of this study’s analysis, particularly from an environmental justice perspective, is in accounting for embodied emissions in the exposure assessment. The results show the importance of accounting for supply chain exposure effects when assessing the impacts of any infrastructure system. The embodied sources contribute to up to 7.5% of all damages. Furthermore, supply chain effects not only increase the absolute exposure of a population, but the exposure disparity of minority and low-income groups too since the target industrial facilities are often located near these populations.^55^

### 5.3 Mitigation Scenarios for Marine Ports

The three broad categories of mitigation strategies explored in this study offer some direction for further inquiry and research. Of the mitigation strategies explored in the study, drayage truck electrification is the most concretely defined strategy that also has growing public policy interest. While we analyze the impacts from reducing or eliminating port operation through unspecified means, emissions from OGVs and other harbor craft could be mitigated through electrification. There are important practical implications to electrifying port operations that should be considered, such as the intersections between connecting OGVs/harbor craft to onshore power or other fuel sources, and making sure that the Port operation electrification infrastructure does not interfere with drayage trucking infrastructure. A future direction for research should investigate the embodied impacts of adding all the necessary charging infrastructure needed to enact these mitigation strategies.

### 5.4 Study Limitations

An important potential limitation of this study is the implication of system boundary selection. For example, what are the effects on exposure when we limit the emission sources from drayage truck operations, or exclude the embodied emissions from fuel used in powering the OGVs and harbor craft or the railcars? The answer to this question largely depends upon the location of where these additional emissions occur. Inclusion, for example, of the construction emissions associated with maintaining the Port’s surface, would further amplify the impacts we already report. However, a limitation of this work is that the boundaries of the exposure domain mean that we fail to capture the impacts from other upstream emissions (e.g., OGV bunker fuel or the shipped goods themselves). Those other upstream emissions would have a greater exposure on the populations living in closer proximity to their respective production. To expand the boundaries of an exposure assessment of a complex civil infrastructure system, such as a marine port, further and more accurately, there need to be higher quality and specific location-based emissions inventories of upstream and on-site goods/activities, which are data missing from most life-cycle inventories.

Another limitation of this work is that we do not examine which mitigation strategies are the most impactful in reducing exposure burdens for specific groups. However, it is possible to reach some likely conclusions without performing further analysis. For the strategies that focus on reducing port-related emissions (e.g., 20% reduction in port cargo handling equipment), the population’s exposure will be reduced in the same scaled manner because the ISRM models linearized marginal changes in exposure concentration. Reductions in emissions from material production facilities and material deliveries might yield different outcomes. It is important to model the differential impact of mitigation strategies on different demographics, as has been done for power plants,^69^ transportation systems, and other built infrastructure,^70^ so that benefits and burdens are distributed equitably.

A further limitation of this study is the assumption we make regarding how much of the Port’s pavement area is maintained each year. We assume that, on average, 2% to 5% of the Port’s surface would be maintained and thus require new materials to be delivered and placed on site. As the Port Authority does not publicly disclose how frequently it resurfaces the Port’s area, we can only make a reasonable assumption and present a range to reduce the uncertainty with this assumption. However, without further confirmation about the Port’s resurfacing routine, the embodied emissions and resulting exposure impacts from material production and delivery to site could be either overestimated or underestimated. It will be valuable for future exposure assessments to have a more certain estimation of how much and how often materials are consumed during maintenance.

### 5.5 Future Directions and Calls to Action

The general methodological approaches, data sources, and assumptions detailed in this study can be adapted by stakeholders in new locations to investigate the equity considerations of infrastructure system emissions and related exposure. Including embodied emissions in an exposure assessment, from a policy and community effort planning approach, should assist stakeholders in identifying more sources of emissions that they need to manage and mitigate. The methods and results of this study build upon prior efforts that incorporate life-cycle thinking and quantitative indicators to promote environmental justice in civil infrastructure systems.^71^

Our study supports recent recommendations for improving existing air quality planning efforts by incorporating quantitative metrics of disparity and tackling pollution disparity at a location-specific spatial scale. Emerging efforts to quantify environmental justice, such as the Biden-Harris Administration’s Justice 40 initiative, are currently not likely effective at alleviating exposure disparities by race/ethnicity, which are typically greater than by income level or disadvantaged community designation.^56^ Location-specific emission reduction strategies have also been found to be more effective than prevailing sector- or region-specific reduction efforts at reducing disparities in exposure among racial-ethnic groups and are more efficient at reducing population-average concentrations.^72^ We add to these recommendations for air quality planning that it is important to treat a location-based emission source, such as the Port of Oakland, as a complex system that has colocated upstream and downstream sources. A systems-based approach is critical for identifying the specific emission sources and mitigation strategies for reducing both absolute and relative exposure disparities.

## Supporting Information Available

(1) An extended literature review on air pollution from the Port of Oakland in West Oakland, (2) description of Port of Oakland emission sources, (3) all exposure mitigation strategies descriptions and results, (4) ranking of sources by exposure disparity for each racial demographic and income quintiles, and (5) monetized exposure damages results for all mitigation strategies (PDF)
