# Supplementary material for: Environmental Justice and Systems Analysis for Air Quality Planning in the Port of Oakland in California
Source: Environ Sci Technol. 2024 May 2;58(19):8135–48. doi: 10.1021/acs.est.3c07728 (PMC11097628; doi:10.1021/acs.est.3c07728)
Supplement: Supplementary file 1 — es3c07728_si_001.pdf [file es3c07728_si_001.pdf]

Environmental Justice and Systems Analysis for Air Quality Planning in the Port of Oakland in California

(Supporting Information)

Fiona Greer[†]*, *Ahmad Bin Thaneya*[†] *and Arpad Horvath*[†]

[†] Department of Civil and Environmental Engineering, University of California, Berkeley
*Corresponding author: fionagreer@berkeley.edu

Pages: 13

Figures: 2

Tables: 4

Background

1. Port of Oakland – Additional Literature Review

An early mobile monitoring study showed that diesel particulate matter concentrations along high-trafficked roadways near the Port of Oakland were 5 times higher than the community's average.[1] One study examined the effectiveness of regulations (e.g., mandatory diesel particle filters on trucks, cleaner fleets) on drayage trucks in the Port, estimating that mitigation efforts reduced black carbon (BC) and nitrogen oxide (NO_x) emission factors by 50% and 40%, respectively.[2] Analysis of previous regulations on drayage trucks operating at the Port indicated that regulations resulted in a 75% decrease in primary particulate matter (PM) emissions from trucks.[3] Further efforts estimated that drayage trucks equipped with diesel particle filters and selective catalytic reduction systems can greatly reduce NO_x, BC, and PM emission factors by 69%, 92%, and 66%, respectively.[4] Another study assessed how shifting the Port's freight operations to night hours can influence overall $PM_{2.5}$ concentrations and exposure. Shifting the timing of Port operations can lead to increases in ambient concentrations of $PM_{2.5}$ due to the atmospheric boundary layer being more stable at nighttime, which reduces mixing and increases concentrations from pollutant emissions. The study found that moving freight operations to the nighttime in the Port of Oakland lead to no change in reducing $PM_{2.5}$ exposure in nearby areas.[5]

West Oakland is home to a large network of air quality monitors due to the high concentration of variable emission sources within its domain. Recent studies have leveraged this extensive monitoring network to capture the spatiotemporal variability of BC concentrations in West Oakland[6] and develop a spatiotemporal model capable of accurately predicting BC concentrations in areas in West Oakland that lack monitors.[7] West Oakland has been utilized to test and exhibit advanced high-resolution air quality mapping techniques using Google Street

View vehicles with pollutant concentration measuring instruments.[8,9] The vehicle mapping techniques demonstrated how urban air pollution gradients can be measured at a very fine scale while also providing measurement resolutions that are finer than those of urban ambient monitors. The fine-scale hyperlocal measurements in West Oakland have allowed for more accurate health impact assessments in the area.[10] Other studies have also employed Chemical Transport Models to analyze air quality impacts in West Oakland. Researchers used the Weather Research and Forecasting model to determine BC concentrations in West Oakland, with a major emphasis placed on source apportionment due to the high number of varying emission sources in the area.[11,12] Both studies predict BC concentrations at reasonably accurate levels and are in agreement with monitoring measurements. They find that on-road diesel trucks are the major contributors to population-weighted BC concentrations, followed by marine and rail sources.

Community participatory research is often regarded as a means to build community power since it involves those that are mostly affected by the study's issues in the study design, execution, interpretation, and later decision-making. Community-based engagement in environmental justice related issues in West Oakland have long been in effect, and a study sought to explore how power and privilege issues have played out in partnership between community partnerships with academic organizations in order to improve the nature and efficacy of such partnerships.[13] The study showed that despite such partnerships being sometimes riddled with power paradigms that shift with location and context, the benefits of such collaborations strengthen credibility to community-groups due to having an independent research partner, especially in technical or politically contentious decision-making processes. They also can provide autonomy to the community-groups to lead such efforts independently and manage future studies themselves while also promoting mutual learning between both organizations.

Other efforts in West Oakland such as artistic activism have been shown to be powerful tools for inclusive engagement and participation of local communities, especially women and youth of color, in environmental related issues. This was demonstrated in a study where researchers analyzed the relations between community-based activism in West Oakland against the construction of a coal-export terminal in the Port of Oakland.[14] Such engagement practices help raise awareness to environmental issues, and in turn promote participation in important decision making and policy-making practices.

2. Tabulated Data and Results

Table S1: Description of sources included in Port of Oakland system boundary.

Source	Description
Ocean-going Vessels (OGV)	• OGV (Cruise Zone): Ships enter the cruise zone from three shipping channels in the Pacific Ocean. Once the ship reaches the Sea Buoy, they reduce speeds and enter the Reduced Speed Zone (RSZ)<br>• OGV (RSZ): Ships enter the RSZ once they pass the Sea Buoy. Once they pass the west span of the Bay Bridge, commercial harbor craft (CHC) assist in tugging the OGV into berth at the Port of Oakland.<br>• Within the harbor area of the Port of Oakland, OGV activities include maneuvering, shifting, berth operations, and anchorage
Commercial Harbor Craft (CHC)	Includes any assist tug operations with OGV and dredging activities associated with maintaining the channel and berth integrity
Port of Oakland Operations	• Cargo Handling Equipment (CHC): Equipment including cranes and forklifts used in transferring cargo containers within the Port of Oakland<br>• Any off-road equipment used in the construction and maintenance of the Port<br>• Railyard activities that happen within the Port
Drayage Trucks	• Emissions from within terminal idling and driving as well as driving from terminal to freeway entrances<br>• We do not account for emissions beyond the freeway entrance
Rail	Emissions associated with rail operations in the Union Pacific Railyard

Source	Description
Materials from Port Maintenance	Concrete<br>Cement<br>Asphalt<br>Aggregate<br>Bitumen
Fuel	Fuel from operating the drayage trucks, delivery of maintenance materials

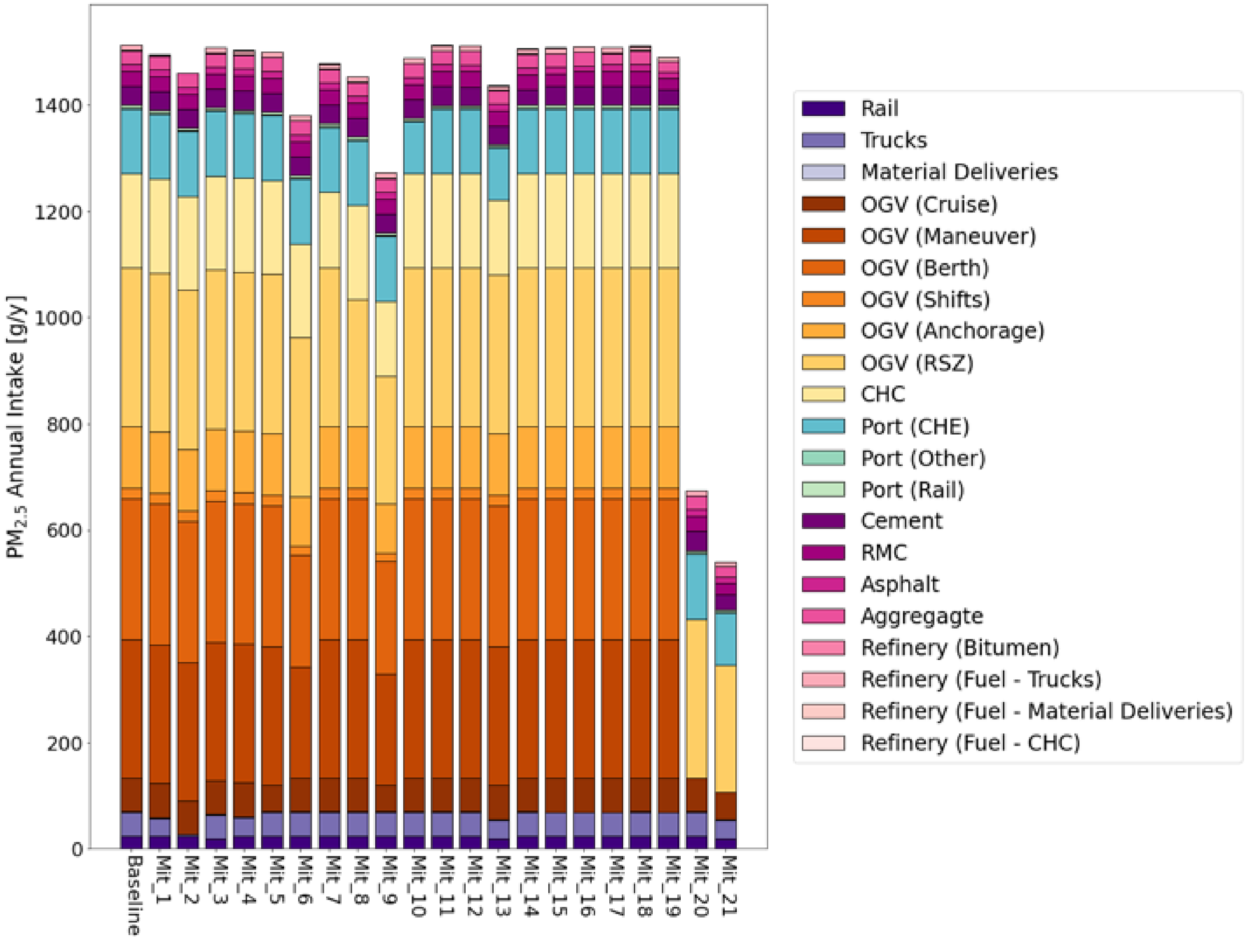

Figure S1: Annual $PM_{2.5}$ intake for the Port of Oakland under the 5% resurfacing scenario. Descriptions of mitigation strategies are included in the main manuscript.

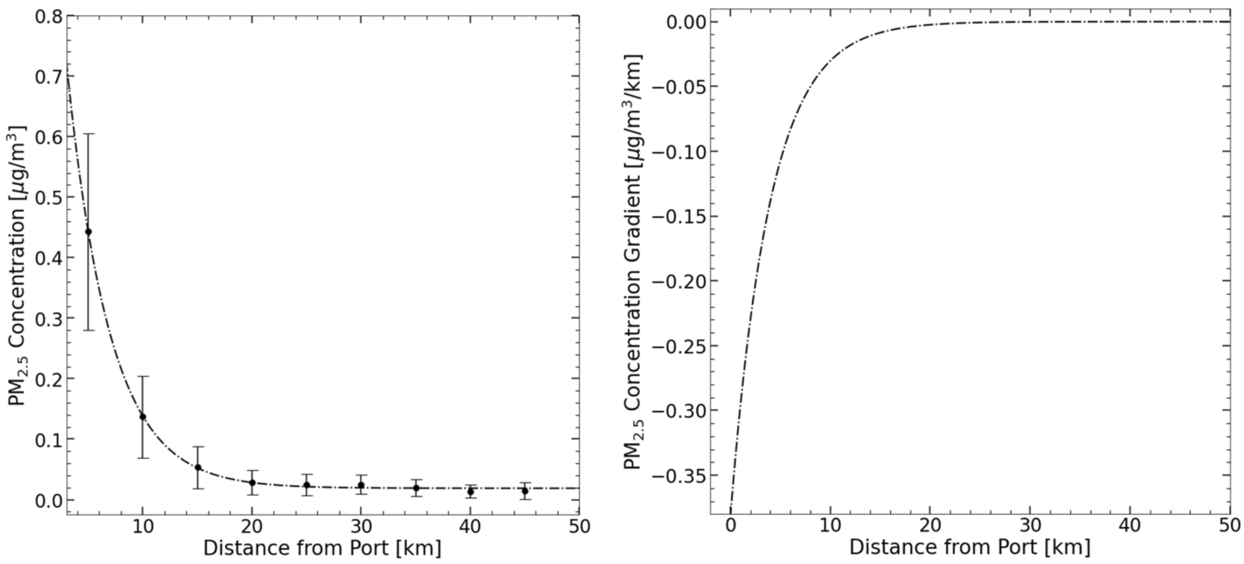

Figure S2: (left) $PM_{2.5}$ concentrations due to Port of Oakland related emissions as a function of distance from the Port. Each data point shows aggregated average $PM_{2.5}$ concentrations in census tracts around the shown distances, while the error bars indicate the standard deviation of concentrations within those census tracts. A trendline (following an exponential decay function) is fit through the data points and shows that concentration gradients are highest at distances <20km to the Port and concentrations quickly level-off beyond 20 km. (right) Plotted $PM_{2.5}$ concentration gradient showing the rate at which $PM_{2.5}$ concentrations decrease given the distance from the Port.

Table S2. Ranking of sources by exposure disparity for each racial demographic. Positive values indicate a greater-than-average exposure from that source; negative values indicate a lower-than-average exposure. For example, the Black population experiences 3.5 times greater-than-average $PM_{2.5}$ exposure from aggregate facilities compared to all people within the San Francisco Bay Area.

	**White**		**Black**		**Asian**		**Hispanic**		**Pacific Islander**		**Native American**	
Ranking of Sources by Exposure Disparity Percentage	OGV - Cruise	1.46%	Agg.	250%	Cem.	32.40%	Agg.	67.00%	Cem.	128%	Asph.	99.00%
	OGV - RSZ	0.54%	Rail (UP)	222%	OGV - Cruise	-0.20%	Bitumen	43.80%	Agg.	44.60%	Agg.	49.90%
	Drayage Trucks - Fuel	-0.16%	Asph.	194%	OGV - RSZ	-0.60%	Asph.	43.80%	OGV - Cruise	9.34%	Cem.	26.00%
	CHC - Fuel	-0.16%	RMC	184%	CHC	-15.80%	Deliveries - Fuel	35.60%	Asph.	-3.99%	CHC	25.70%
	Material Deliveries - Fuel	-0.16%	Port - Other	171%	OGV - Berths	-16.50%	Drayage Trucks - Fuel	35.60%	OGV - Berths	-7.55%	RMC	25.40%
	Bitumen	-2.45%	Drayage Trucks	170%	OGV - Anchorage	-16.60%	CHC - Fuel	35.60%	OGV - Anchorage	-7.80%	Rail (UP)	24.80%
	Material Deliveries	-8.52%	Material Deliveries	170%	OGV - Maneuvers	-16.70%	Cem.	14.00%	OGV - Shifts	-7.91%	OGV - Maneuvers	24.50%
	Drayage Trucks	-8.52%	Port - Rail	170%	OGV - Shifts	-16.70%	OGV - Cruise	-6.71%	OGV - Maneuvers	-8.34%	OGV - Shifts	24.40%
	OGV - Shifts	-9.30%	Port - CHE	167%	RMC	-18.00%	OGV - Berths	-13.30%	CHC	-8.93%	OGV - Anchorage	24.40%
	OGV - Anchorage	-9.34%	CHC	151%	Port - Other	-19.70%	OGV - Anchorage	-13.40%	CHE	-11.80%	OGV - Berths	24.30%
	OGV - Berths	-9.38%	OGV - Maneuvers	144%	Port - Rail	-20.00%	OGV - Shifts	-13.40%	Port - Rail	-11.90%	Port - Rail	23.50%
	OGV - Maneuvers	-9.40%	OGV - Shifts	143%	CHE	-20.20%	OGV - Maneuvers	-13.80%	Port - Other	-12.00%	Port - Other	23.50%
	CHE	-9.57%	OGV - Anchorage	142%	Rail (UP)	-21.60%	CHC	-15.00%	Material Deliveries	-12.80%	CHE	23.10%
	Port - Rail	-10.00%	OGV - Berths	142%	Drayage Trucks	-22.50%	CHE	-17.00%	Drayage Trucks	-12.80%	Drayage Trucks	21.30%
	Port - Other	-10.30%	Bitumen	91.00%	Material Deliveries	-22.50%	Port - Rail	-17.30%	RMC	-16.00%	Material Deliveries	21.30%
	CHC	-10.70%	Deliveries - Fuel	82.80%	Asph.	-34.80%	Trucks	-17.40%	Rail (UP)	-16.40%	CHC - Fuel	-4.92%

	White		Black		Asian		Hispanic		Pacific Islander		Native American	
	RMC	-11.40%	CHC - Fuel	82.80%	CHC - Fuel	-37.40%	Material Deliveries	-17.40%	CHC - Fuel	-18.40%	Drayage Trucks - Fuel	-4.92%
	Rail (UP)	-14.30%	Drayage Trucks - Fuel	82.80%	Drayage Trucks - Fuel	-37.40%	Port - Other	-17.80%	Drayage Trucks - Fuel	-18.40%	Deliveries - Fuel	-4.92%
	Cem.	-20.80%	OGV - RSV	38.00%	Deliveries - Fuel	-37.50%	RMC	-20.90%	Deliveries - Fuel	-18.40%	Bitumen	-6.74%
	Asph.	-27.10%	OGV - Cruise	-2.79%	Agg.	-38.40%	Rail (UP)	-23.80%	Bitumen	-23.10%	OGV - RSZ	-7.83%
	Agg.	-37.30%	Cem.	-22.70%	Bitumen	-38.80%	OGV - RSZ	-25.30%	OGV - RSZ	-27.10%	OGV - Cruise	-10.90%

Table S3. Ranking of sources by exposure disparity for each income quintile. Positive values indicate a greater-than-average exposure from that source; negative values indicate a lower-than-average exposure. For example, the Q1 income quintile experiences 2.97 times greater-than-average $PM_{2.5}$ exposure from aggregate facilities compared to all people within the San Francisco Bay Area.

	Q1		Q2		Q3		Q4		Q5	
Ranking of Sources by Exposure Disparity Percentage	Agg.	197%	Deliveries - Fuel	18.50%	Cem.	15.90%	Cem.	17.10%	Cem.	25.50%
	Asph.	182%	Drayage Trucks - Fuel	18.50%	OGV - Cruise	3.98%	OGV - Cruise	6.96%	OGV - RSZ	10.70%
	Rail (UP)	147%	CHC - Fuel	18.50%	OGV - RSZ	-13.10%	OGV - RSZ	-12.90%	OGV - Cruise	3.45%
	Bitumen	128%	Bitumen	16.50%	OGV - Berths	-17.70%	Drayage Trucks - Fuel	-40.10%	OGV - Berths	-33.60%
	RMC	124%	RMC	7.68%	OGV - Anchorage	-17.70%	CHC - Fuel	-40.10%	OGV - Anchorage	-33.70%
	Port - Other	116%	Rail (UP)	4.52%	OGV - Shifts	-17.70%	Deliveries - Fuel	-40.10%	OGV - Shifts	-33.80%
	Port - Rail	115%	Port - Rail	2.17%	OGV - Maneuvers	-17.80%	OGV - Berths	-46.00%	OGV - Maneuvers	-34.20%
	CHE	113%	Port - Other	2.10%	CHC	-18.60%	OGV - Anchorage	-46.10%	CHC	-36.10%
	CHC	111%	CHE	1.97%	Drayage Trucks - Fuel	-20.00%	OGV - Shifts	-46.20%	Drayage Trucks	-36.20%
	Drayage Trucks	110%	Drayage Trucks	1.96%	CHC - Fuel	-20.00%	OGV - Maneuvers	-46.80%	Material Deliveries	-36.20%
	Material Deliveries	110%	Material Deliveries	1.96%	Deliveries - Fuel	-20.00%	Bitumen	-47.70%	CHE	-37.10%
	Deliveries - Fuel	107%	CHC	0.80%	Material Deliveries	-20.50%	Drayage Trucks	-49.10%	RMC	-37.20%
	CHC - Fuel	107%	OGV - Shifts	0.49%	Drayage Trucks	-20.50%	Material Deliveries	-49.10%	Port - Rail	-37.80%
	Drayage Trucks - Fuel	107%	OGV - Maneuvers	0.49%	CHE	-20.60%	CHE	-50.50%	Port - Other	-38.00%
	OGV - Maneuvers	105%	OGV - Anchorage	0.49%	Port - Rail	-20.90%	CHC	-50.70%	Rail (UP)	-46.00%
	OGV - Shifts	103%	OGV - Berths	0.47%	Port - Other	-21.10%	Asph.	-51.50%	CHC - Fuel	-59.00%
	OGV - Anchorage	103%	Agg.	-5.05%	Bitumen	-26.00%	Port - Rail	-51.80%	Drayage Trucks - Fuel	-59.00%
	OGV - Berths	103%	OGV - Cruise	-6.46%	Rail (UP)	-30.60%	Port - Other	-52.50%	Deliveries - Fuel	-59.00%
	OGV - RSZ	38.30%	Asph.	-8.94%	RMC	-32.30%	RMC	-54.80%	Asph.	-63.30%
	OGV - Cruise	-8.57%	OGV - RSZ	-20.50%	Asph.	-47.30%	Agg.	-58.10%	Bitumen	-63.30%
	Cem.	-17.40%	Cem.	-42.70%	Agg.	-50.10%	Rail (UP)	-65.60%	Agg.	-72.40%

Ranking of Sources by Exposure Disparity Percentage	Q1		Q2		Q3		Q4		Q5	
	Agg.	197%	Deliveries - Fuel	18.50%	Cem.	15.90%	Cem.	17.10%	Cem.	25.50%
	Asph.	182%	Drayage Trucks - Fuel	18.50%	OGV - Cruise	3.98%	OGV - Cruise	6.96%	OGV - RSZ	10.70%
	Rail (UP)	147%	CHC - Fuel	18.50%	OGV - RSZ	-13.10%	OGV - RSZ	-12.90%	OGV - Cruise	3.45%
	Bitumen	128%	Bitumen	16.50%	OGV - Berths	-17.70%	Drayage Trucks - Fuel	-40.10%	OGV - Berths	-33.60%
	RMC	124%	RMC	7.68%	OGV - Anchorage	-17.70%	CHC - Fuel	-40.10%	OGV - Anchorage	-33.70%
	Port - Other	116%	Rail (UP)	4.52%	OGV - Shifts	-17.70%	Deliveries - Fuel	-40.10%	OGV - Shifts	-33.80%
	Port - Rail	115%	Port - Rail	2.17%	OGV - Maneuvers	-17.80%	OGV - Berths	-46.00%	OGV - Maneuvers	-34.20%
	CHE	113%	Port - Other	2.10%	CHC	-18.60%	OGV - Anchorage	-46.10%	CHC	-36.10%
	CHC	111%	CHE	1.97%	Drayage Trucks - Fuel	-20.00%	OGV - Shifts	-46.20%	Drayage Trucks	-36.20%
	Drayage Trucks	110%	Drayage Trucks	1.96%	CHC - Fuel	-20.00%	OGV - Maneuvers	-46.80%	Material Deliveries	-36.20%
	Material Deliveries	110%	Material Deliveries	1.96%	Deliveries - Fuel	-20.00%	Bitumen	-47.70%	CHE	-37.10%
	Deliveries - Fuel	107%	CHC	0.80%	Material Deliveries	-20.50%	Drayage Trucks	-49.10%	RMC	-37.20%
	CHC - Fuel	107%	OGV - Shifts	0.49%	Drayage Trucks	-20.50%	Material Deliveries	-49.10%	Port - Rail	-37.80%
	Drayage Trucks - Fuel	107%	OGV - Maneuvers	0.49%	CHE	-20.60%	CHE	-50.50%	Port - Other	-38.00%
	OGV - Maneuvers	105%	OGV - Anchorage	0.49%	Port - Rail	-20.90%	CHC	-50.70%	Rail (UP)	-46.00%
	OGV - Shifts	103%	OGV - Berths	0.47%	Port - Other	-21.10%	Asph.	-51.50%	CHC - Fuel	-59.00%
	OGV - Anchorage	103%	Agg.	-5.05%	Bitumen	-26.00%	Port - Rail	-51.80%	Drayage Trucks - Fuel	-59.00%
	OGV - Berths	103%	OGV - Cruise	-6.46%	Rail (UP)	-30.60%	Port - Other	-52.50%	Deliveries - Fuel	-59.00%
	OGV - RSZ	38.30%	Asph.	-8.94%	RMC	-32.30%	RMC	-54.80%	Asph.	-63.30%
	OGV - Cruise	-8.57%	OGV - RSZ	-20.50%	Asph.	-47.30%	Agg.	-58.10%	Bitumen	-63.30%
	Cem.	-17.40%	Cem.	-42.70%	Agg.	-50.10%	Rail (UP)	-65.60%	Agg.	-72.40%

Table S4. Exposure damages for baseline and mitigation strategies. A negative percentage change indicates a reduction in monetized exposure damages. Three significant digits are shown to make distinctions in the ranges.

**Description**	**Exposure Damages ($M/year) 2% Scenario**	**Exposure Damages ($M/year) - 5% Scenario**
Baseline	100 – 260	110 – 270
Truck 2045 Scenario	1.08% – 1.10%	1.05% – 1.06%
Truck Electrification	2.95% – 3.02%	2.89% – 2.97%
Rail Reduction (20%)	0.26% – 0.28%	0.25% – 0.27%
Trucking Reduction (20%)	0.61% – 0.63%	0.59% – 0.61%
OGV Cruise Reduction (20%)	0.78% – 0.76%	0.75% – 0.73%
OGV In-Harbor Reduction (20%)	10.39% – 10.70%	10.00% – 10.30%
CHC Reduction (20%)	1.98% – 2.05%	1.91% – 1.98%
OGV RSZ Reduction (20%)	3.63% – 3.64%	3.50% – 3.51%
OGV + CHC All Reduction (20%)	16.79% – 17.14%	16.20% – 16.60%
Port CHE Reduction (20%)	1.36% – 1.41%	1.31% – 1.37%
Port Other Reduction (20%)	0.03% – 0.03%	0.03% – 0.03%
Port Rail Reduction (20%)	0.05% – 0.05%	0.05% – 0.05%
Port + CHC All Reduction (20%)	4.29% – 4.45%	4.14% – 4.29%
Cement Reduction (20%)	0.16% – 0.15%	0.37% – 0.36%
RMC Reduction (20%)	0.13% – 0.13%	0.30% – 0.32%
Asphalt Reduction (20%)	0.06% – 0.06%	0.14% – 0.15%
Aggregate Reduction (20%)	0.12% – 0.12%	0.29% – 0.29%
Refineries Reduction (20%)	0.13% – 0.13%	0.13% – 0.13%
All Facility Reduction (20%)	0.59% – 0.59%	1.24% – 1.25%
OGV Harbor + CHC Emission Elimination	62.81% – 63.70%	60.60% – 61.50%
Combine All	70.19% – 70.96%	68.40% – 69.20%

References

(1) Fujita, E. M.; Campbell, D. E.; Patrick Arnott, W.; Lau, V.; Martien, P. T. Spatial Variations of Particulate Matter and Air Toxics in Communities Adjacent to the Port of Oakland. *J. Air Waste Manag. Assoc.* **2013**, *63* (12), 1399–1411.

(2) Dallmann, T. R.; Harley, R. A.; Kirchstetter, T. W. Effects of Diesel Particle Filter Retrofits and Accelerated Fleet Turnover on Drayage Truck Emissions at the Port of Oakland. *Environ. Sci. Technol.* **2011**, *45* (24), 10773–10779.

(3) Kuwayama, T.; Schwartz, J. R.; Harley, R. A.; Kleeman, M. J. Particulate Matter Emissions Reductions Due to Adoption of Clean Diesel Technology at a Major Shipping Port. *Aerosol Sci. Technol.* **2013**, *47* (1), 29–36.

(4) Preble, C. V.; Dallmann, T. R.; Kreisberg, N. M.; Hering, S. V.; Harley, R. A.; Kirchstetter, T. W. Effects of Particle Filters and Selective Catalytic Reduction on Heavy-Duty Diesel Drayage Truck Emissions at the Port of Oakland. *Environ. Sci. Technol.* **2015**, *49* (14), 8864–8871.

(5) Sathaye, N.; Harley, R.; Madanat, S. Unintended Environmental Impacts of Nighttime Freight Logistics Activities. *Transp. Res. Part Policy Pract.* **2010**, *44* (8), 642–659. https://doi.org/10.1016/j.tra.2010.04.005.

(6) Caubel, J. J.; Cados, T. E.; Preble, C. V.; Kirchstetter, T. W. A Distributed Network of 100 Black Carbon Sensors for 100 Days of Air Quality Monitoring in West Oakland, California. *Environ. Sci. Technol.* **2019**, *53* (13), 7564–7573. https://doi.org/10.1021/acs.est.9b00282.

(7) Wai, T. H.; Apte, J. S.; Harris, M. H.; Kirchstetter, T. W.; Portier, C. J.; Preble, C. V.; Roy, A.; Szpiro, A. A. Insights from Application of a Hierarchical Spatio-Temporal Model to an Intensive Urban Black Carbon Monitoring Dataset. *Atmos. Environ.* **2022**, *277*, 119069. https://doi.org/10.1016/j.atmosenv.2022.119069.

(8) Apte, J. S.; Messier, K. P.; Gani, S.; Brauer, M.; Kirchstetter, T. W.; Lunden, M. M.; Marshall, J. D.; Portier, C. J.; Vermeulen, R. C. H.; Hamburg, S. P. High-Resolution Air Pollution Mapping with Google Street View Cars: Exploiting Big Data. *Environ. Sci. Technol.* **2017**, *51* (12), 6999–7008. https://doi.org/10.1021/acs.est.7b00891.

(9) Messier, K. P.; Chambliss, S. E.; Gani, S.; Alvarez, R.; Brauer, M.; Choi, J. J.; Hamburg, S. P.; Kerckhoffs, J.; LaFranchi, B.; Lunden, M. M.; Marshall, J. D.; Portier, C. J.; Roy, A.; Szpiro, A. A.; Vermeulen, R. C. H.; Apte, J. S. Mapping Air Pollution with Google Street View Cars: Efficient Approaches with Mobile Monitoring and Land Use Regression. *Environ. Sci. Technol.* **2018**, *52* (21), 12563–12572. https://doi.org/10.1021/acs.est.8b03395.

(10) Goin, D. E.; Sudat, S.; Riddell, C.; Morello-Frosch, R.; Apte, J. S.; Glymour, M. M.; Karasek, D.; Casey, J. A. Hyperlocalized Measures of Air Pollution and Preeclampsia in Oakland, California. *Environ. Sci. Technol.* **2021**, *55* (21), 14710–14719. https://doi.org/10.1021/acs.est.1c02151.

(11) Joe, D. K.; Zhang, H.; DeNero, S. P.; Lee, H.-H.; Chen, S.-H.; McDonald, B. C.; Harley, R. A.; Kleeman, M. J. Implementation of a High-Resolution Source-Oriented WRF/Chem Model at the Port of Oakland. *Atmos. Environ.* **2014**, *82*, 351–363. https://doi.org/10.1016/j.atmosenv.2013.09.055.

(12) Hamilton, S. D.; Harley, R. A. High-Resolution Modeling and Apportionment of Diesel-Related Contributions to Black Carbon Concentrations. *Environ. Sci. Technol.* **2021**, *55* (18), 12250–12260. https://doi.org/10.1021/acs.est.1c03913.

(13) Garzón, C.; Beveridge, B.; Gordon, M.; Martin, C.; Matalon, E.; Moore, E. Power, Privilege, and the Process of Community-Based Participatory Research: Critical Reflections on Forging an Empowered Partnership for Environmental Justice in West Oakland, California. *Environ. Justice* **2013**, *6* (2), 71–78. https://doi.org/10.1089/env.2012.0039.

(14) Sanz, T.; Rodriguez-Labajos, B. Does Artistic Activism Change Anything? Strategic and Transformative Effects of Arts in Anti-Coal Struggles in Oakland, CA. *Geoforum* **2021**, *122*, 41–54. https://doi.org/10.1016/j.geoforum.2021.03.010.
